# Ecologically sustainable human exploitation of the Gran Dolina TD10.2 bison (Sierra de Atapuerca, Spain)

**DOI:** 10.1038/s41598-025-01928-w

**Published:** 2025-07-02

**Authors:** Guillermo Rodríguez-Gómez, Antonio Rodríguez-Hidalgo, Palmira Saladié, Jan van der Made, Juan Marín, Andreu Ollé, Marina Mosquera, José María Bermúdez de Castro, Juan Luis Arsuaga, Eudald Carbonell

**Affiliations:** 1 Departamento de Geodinámica, Estratigrafía y Paleontología, Universidad Complutense de Madrid, C/José Antonio Novais 12, 28040 Madrid, Spain; 2 Centro UCM-ISCIII de Evolución y Comportamiento Humanos, Avd/Monforte de Lemos, 5, Pabellón 14, 28029 Madrid, Spain; 3 Instituto de Arqueología-Mérida, Consejo Superior de Investigaciones Científicas (CSIC-Junta de Extremadura), Plaza de España 15, 06800 Mérida, Spain; 4 Institut Català de Paleoecologia Humana i Evolució Social (IPHES-CERCA), Zona Educacional 4, Campus Sescelades URV (Edifici W3), 43007 Tarragona, Spain; 5 Departament d’Història i Història de l’Art, Universitat Rovira i Virgili, Avinguda de Catalunya 35, 43003 Tarragona, Spain; 6 Unit Associated with CSIC, Departamento de Paleobiología, Museo Nacional de Ciencias Naturales (CSIC), Calle José Gutiérrez Abascal, 2, 28006 Madrid, Spain; 7 Consejo Superior de Investigaciones Científicas, Museo Nacional de Ciencias Naturales, C. José Gutiérrez Abascal 2, 28006 Madrid, Spain; 8 Departamento de Prehistoria y Arqueología, Universidad Nacional de Educación a Distancia (UNED), Paseo Senda del Rey, 7, 28040 Madrid, Spain; 9 Histoire Naturelle des Humanités Préhistoriques (HNHP), UMR 7194, CNRS, UPVD, Muséum National d’Histoire Naturelle, Paris, France; 10 Centro Nacional de Investigación Sobre la Evolución Humana (CENIEH), Paseo de la Sierra de Atapuerca 3, 09002 Burgos, Spain

**Keywords:** Lower Paleolithic, Subsistence strategies, Mass kill, Communal hunting, Mortality profiles, Life tables, Body mass, Energy extraction, Pre-Neanderthals, Palaeoecology, Animal behaviour, Behavioural ecology, Population dynamics

## Abstract

There is evidence that communal hunting of bison was a practice that occurred from the Middle Pleistocene to historic times and was also observed among Indigenous Americans. Within the subsistence strategies of Pleistocene humans, communal hunting was part of their range of behaviors. The level TD10.2-BB of the Gran Dolina in the Sierra de Atapuerca preserves a fossil record of this practice, with remains of at least 60 bison of different age classes. This study aims to evaluate the hypothesis that human exploitation of these bison was sustainable. To this end, we analyzed the mortality pattern of bison from TD10.2-BB through dental remains using ternary diagrams and life tables. We also used allometric equations to estimate the mean body mass of the bison population and the potential energy yield they could have provided. Our results suggest a catastrophic mortality profile for the TD10.2-BB bison, with individuals of all age classes and no selective bias toward specific age classes. The life table derived from this profile suggests a growing bison population, indicating that human exploitation could have been sustained without causing a population collapse. Furthermore, our results suggest that bison would provide meat and energy resources to support large groups of humans for several days. While the high protein content of bison carcasses may have limited their full utilization, the availability of other resources in the ecosystem likely facilitated the optimal use of bison.

**Supplementary Information:**

The online version contains supplementary material available at 10.1038/s41598-025-01928-w.

## Introduction

Zooarchaeological evidence shows that the bison (*Bison* spp.) was one of the large ungulates frequently hunted by Paleolithic foragers in the Holarctic region for meat, fat, and other resources^1–3^. In Eurasia, especially during the Late Pleistocene, the steppe bison (*Bison priscus*) was a crucial food source for Paleolithic populations. Their skeletal remains often appear alongside those of cervids and equids, indicating regular hunting patterns^1,4^. In North America, the importance of large prey in Indigenous economies has been debated^5^. Nevertheless, bison (*B. antiquus*, *B. bison*) was essential to subsistence strategies from the Late Pleistocene until the nineteenth century^6^. Beyond its economic significance, the bison also held considerable cultural importance. In Western Eurasia, the presence of bison in Upper Paleolithic parietal and portable art underscores its central role in the cosmology of early human populations^7^. In the American context, ethnohistorical records and Indigenous community traditions reflect the bison’s significance up to the present day^8^.

The mass killing of bison, particularly in North America, represents a singular hunting strategy developed by Indigenous peoples. This technique involved the coordinated hunting of multiple bison in a single event and relied on natural traps such as cliffs or corrals, to which herds were driven, often in seasonally organized efforts. These communal hunts involved the entire group, regardless of age, status, or gender, and resulted in large kill sites that are iconic in the archaeology of prehistoric America^2,6^.

In Europe, a similar pattern of bison exploitation can be observed at Middle and Upper Paleolithic sites, where bison remains dominate some faunal assemblages, such as the French sites of Mauran, La Quina, and Coudoulous I, or the German site of Wallertheim, among others^3^. This suggests the use of mass hunting techniques. These sites, which date from Marine Isotope Stage MIS6 to MIS3 (191–57 ka), provide valuable insights into communal hunting dynamics; however, discussions about the social aspects of these activities are limited (^9^ and references therein). One significant example is the bison bone bed layer (TD10.2-BB) at the Gran Dolina site in the Sierra de Atapuerca (Spain). This layer is proposed to be the earliest evidence of mass bison hunting, dating back to MIS11/8^9^ (424–243 ka). The remains of at least 60 individuals of various age groups illustrate this prehistoric mass hunting activity. Analysis of this site reveals a bimodal mortality pattern, with deaths concentrated in late spring/summer and early autumn, reflecting communal hunting efforts and perhaps migratory behavior of this bison population.

Traditionally, it was believed that small-scale hunter-gatherer societies managed and used resources sustainably (e.g.,^10^). However, various theoretical perspectives, ethnographic observations, and archaeological evidence have shown that these societies could exploit certain resources intensively, sometimes leading to depletion^11^.

The methods, techniques, and intensity of mass hunting of complete bison herds during prehistoric times show a significant lack of uniformity. In reality, mass hunting was a distinct phenomenon that has been well-documented through archaeological findings, attracting the attention of many researchers. However, how did the mass hunting affect the TD10.2-BB bison population?

This study aims to determine whether the bison population in TD10.2-BB was sustainably exploited by the human groups that inhabited the area, as well as to assess the energy yield these groups could obtain from the bison. Following Rodríguez-Gómez et al.^12^, we employ various methodological tools (such as mortality profiles and ternary diagrams, life tables, Leslie matrices, and allometric equations to estimate body masses) (see below) to present an innovative and original approach that enhances our understanding of Middle Pleistocene human populations and their subsistence strategies. However, it is essential to consider that this approach requires a fossil record with a high representation of individuals across different age intervals in the mortality profile. Additionally, catastrophic mortality profiles are needed to infer the ecological characteristics of the population, along with remains that enable the estimation of body masses of adult individuals within the group.

## Results

**Fig. 1:**
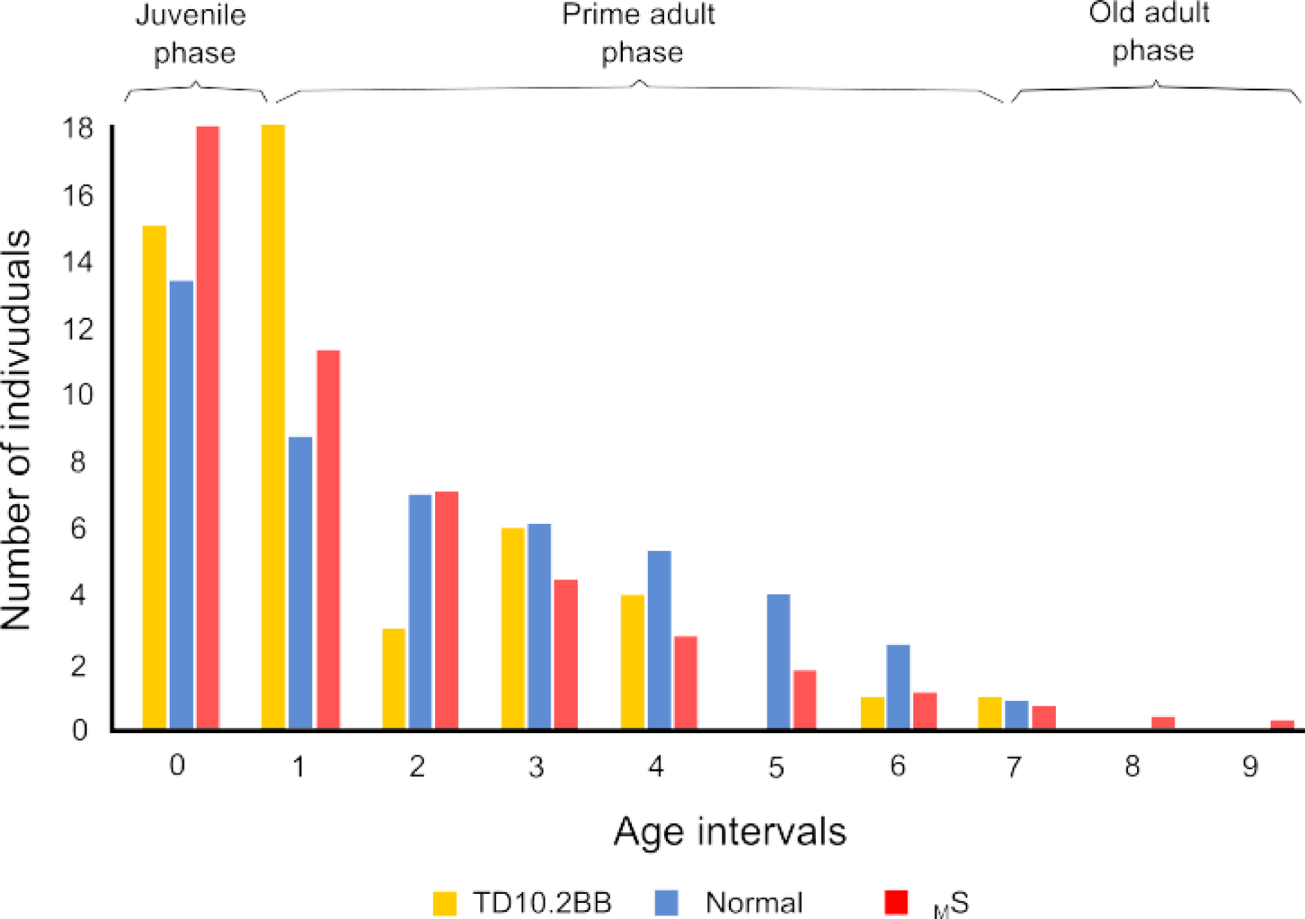
Mortality profiles of bison populations compared in the present study, showing the number of dead individuals by age interval. In order to compare among mortality profiles, we reconstructed profiles of recent bison by Frison and Reher^15^ (Normal) and from Weibull model (S_M_)^16,17^ (see text) with the same total number of individuals as TD10.2-BB population (48 individuals).

The reconstruction of the mortality profile of the TD10.2-BB bison population shows individuals in almost all age classes, except for class 5 (10–12 years), 8 (16–18 years), and 9 (18–20 years) (Fig. 1). Although the maximum ecological longevity we considered is 20 years, the maximum age interval with representation was interval 7 (14–16 years). In terms of the number of individuals, the largest numbers were in the juvenile and prime adult phases, with the juvenile phase showing the largest number prominently, with age interval 1 (2–4 years) with 18 individuals out of the 48 total (37.5%). The first two age intervals (0–4 years) account for 69% of the sample, more than two-thirds.

**Fig. 2:**
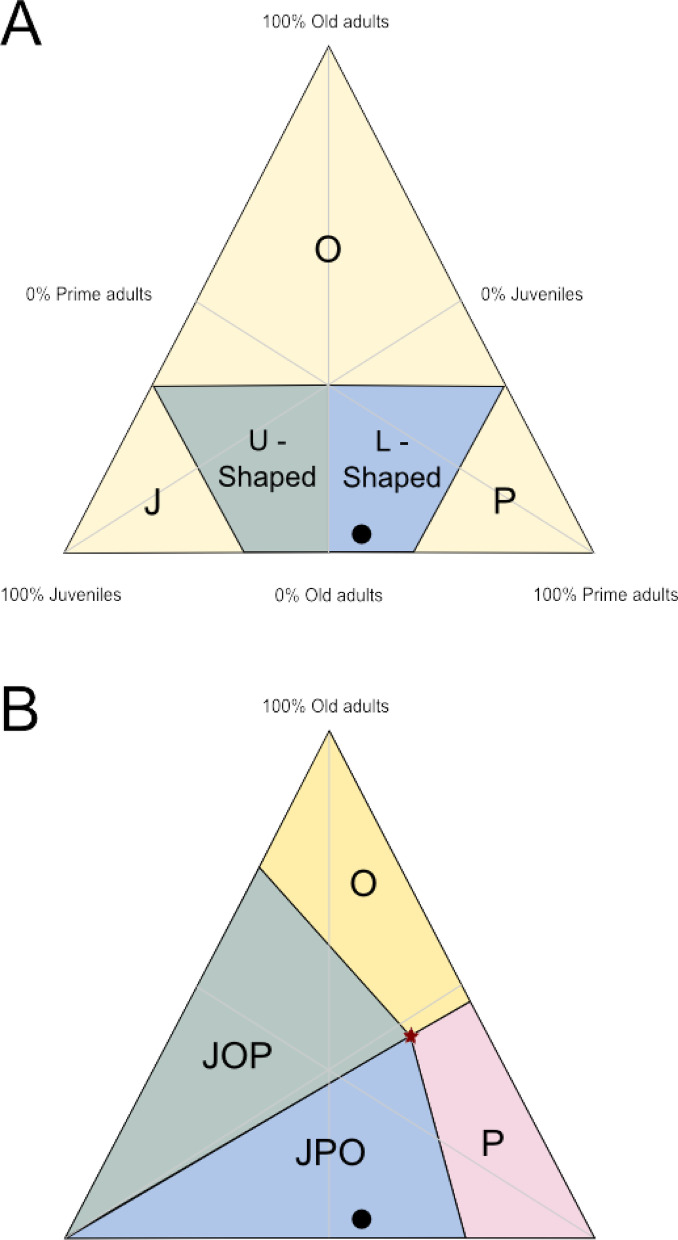
Ternary diagrams showing the percentages of juveniles (J) (42%), adults (P) (54%) and senescent adults (O) (4%) for the TD10.2-BB bison, following the zone definitions of Stiner^13^ (**A**) and Discamps and Costamagno^14^ (**B**). U-shaped region: zone defined by Stiner^13^ for diachronic or attritional mortality profiles; L-shaped region: zone defined by Stiner^13^ for profiles of catastrophic mortality profiles or for living population structures; J: mortality profiles dominated by juvenile individuals; P: mortality profiles dominated by adult individuals; O: mortality profiles dominated by senescent individuals; JPO: living or catastrophic profiles; JOP: diachronic profiles.

The mortality profile seems to tend towards an L-shaped pattern, with the highest number of deaths at the early age intervals, decreasing towards the later intervals. Considering the percentage of individuals by age stage (juvenile, prime adult, and senescent adult), the prime adult stage showed the highest number of individuals with 54%, followed by juvenile with 42% and senescent adult with 4%. Although the prime adult phase had a larger number of individuals, it should be noted that it covers a longer time (3–12 years). Using these percentages for each phase, we constructed the ternary diagrams of Stiner^13^ and Discamps and Costamagno^14^ (Fig. 2) and assessed the overall death pattern of the population. From our results, we obtained that the mortality pattern of TD10.2 bison was located in the catastrophic profile or life structure regions, both for those defined by Stiner^13^ (L-shaped) (Fig. 2A) and Discamps and Costamagno^14^ (JPO) (Fig. 2B). Thus, we did not observe a bias towards young and senescent individuals, as might be expected for hunting strategies with a preference for obtaining the weakest individuals. In the same sense, we did not observe a pattern dominated by prime adults, which has been proposed as a hallmark of human hunting behavior^4^.

Given that the mortality pattern of the TD10.2-BB bison was that expected from a catastrophic or life structure profile, we can reconstruct the life table of this population to understand some of the ecological characteristics (Table 1), the starting information being the proportion of individuals death in each age interval (*d*_*x*_) (see Materials and Method sections). The life table shows that the population had an average mortality rate of 46.8% and a survival rate of 53.2% (Table 1), with class 5 having the highest survival rate and class 4 having the highest mortality rate, apart from class 7. The average value of fertility rate (*m*_*x*_) is 0.58, being maximum (0.88) for classes 2 to 6 (4–14 years) (Table 1). Our results on net growth (*R*_*o*_) for this population show a value > 1 (*R*_*o*_ = 1.228), so the population was growing (Table 1). Regarding the mean generation time, our results indicate a value of 2.169 for *T* (Table 1). Based on these values of *R*_*0*_ and *T*, the TD10.2 bison population provides a value of lambda or the asymptotic growth rate (*λ*) equal to 1.099 (Table 1). Since lambda is the ratio of population size to the previous year, the TD10.2-BB bison population was increasing by 9.9% each year.

**Table 1:** Life table of bison from TD10.2-BB, inferred from the proportion of individuals for each age class (*d*_*x*_).

Age intervals (years) (*X*)	*l*_*x*_	*d*_*x*_	*q*_*x*_	*s*_*x*_	*P*_*i*_	*m*_*x*_	*l*_*x*_*m*_*x*_	*xl*_*x*_*m*_*x*_	Results
0 (0–2)	1.00	0.31	0.31	0.69	0,40	0.00	0.00	0.00	
1 (2–4)	0.69	0.38	0.55	0.45	0,28	0.78	0.54	0.54	
2 (4–6)	0.31	0.06	0.20	0.80	0,13	0.88	0.28	0.55	
3 (6–8)	0.25	0.13	0.50	0.50	0,10	0.88	0.22	0.66	
4 (8–10)	0.13	0.08	0.67	0.33	0,05	0.88	0.11	0.44	
5 (10–12)	0.04	0.00	0.00	1.00	0,02	0.88	0.04	0.18	
6 (12–14)	0.04	0.02	0.50	0.50	0,02	0.88	0.04	0.22	
7 (14–16)	0.02	0.02	1.00	0.00	0,01	0.44	0.01	0.06	
8 (16–18)	0.00	0.00	0.00	0.00	0.00	0.00	0.00	0.00	
9 (18–20)	0.00	0.00	0.00	0.00	0.00	0.00	0.00	0.00	
Average			0.47	0.53		0.56			
Total	2.48	1.00	3.72	4.28			1.23	2.66	
Net reproductive rate (*R*_*0*_)									1.228
Mean generation time (*T*)									2.169
Lambda or asymptotic growth rate (*λ*)*									1.099

The indexes are thoroughly explained in Materials and Methods section.

**Fig. 3:**
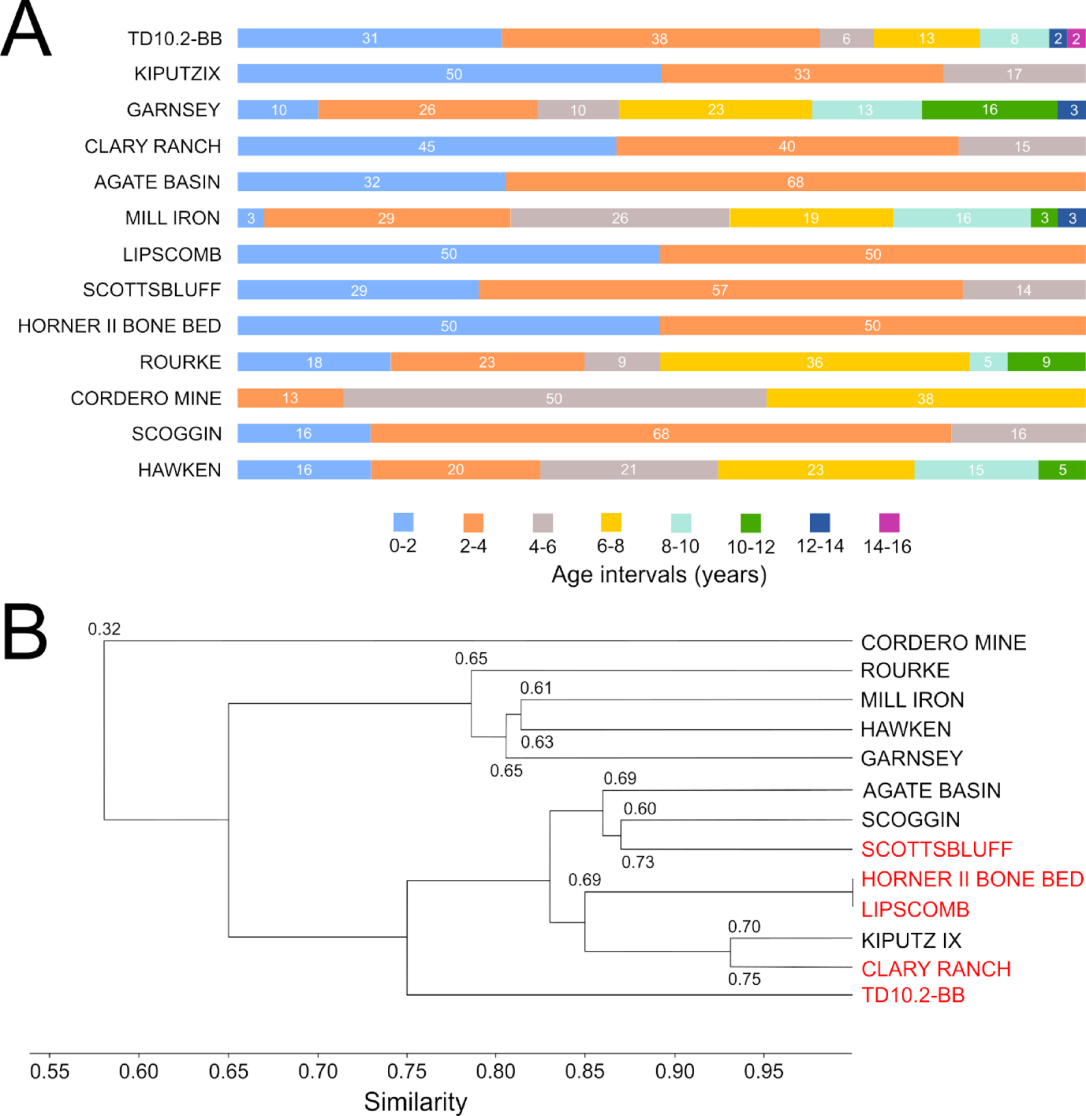
Distribution of the percentage of individuals by age interval for different bison mortality profiles, including those of Gran Dolina level TD10.2-BB (**A**) and similarity dendogram of these mortality profiles (**B**). The dendogram includes the Bray–Curtis similarity index values between TD10.2-BB and each bison assemblage. Kill-butchery sites (Clary Ranch, Horner II Bone Bed, Lipscomb, Scottsbluff, TD10.2-BB) are marked in red to assess the degree of similarity between them. See Table SI2 in the supplemental material for more information.

Comparison of the bison population profile of TD10.2-BB with that of Frison and Reher^15^ showed χ^2^ values (χ^2^ = 10,984; *p* = 0.1409) indicating that there is no significant difference in the distribution of individuals between the two (Fig. 1). This suggests that the TD10.2-BB population had a structure close to that expected by these authors, although it should be noted that the age intervals are not exactly the same length. Regarding the comparison with stable and stationary structures obtained with the Weibulll model^16,17^, we also found no significant differences between most of the analyzed profiles (maximum survival (S_M_) 7.8195, *p* = 0.3478; minimum survival (S_m_) 9.1908, *p* = 0.41985; minimum survival (M_m_) 7.8195, *p* = 0.34878), having these structures lower χ^2^ values than those obtained in the comparison with the structure proposed by Frison and Reher^15^. In addition, we compared the Frison and Reher^15^ structure with those of the Weibull model and obtained lower values than in the comparison with the TD10.2 bison. Figure 1 shows how similar the age profiles reconstructed for the TD10.2 population, the Frison and Reher^15^ population and the maximum survival (S_M_) profile are. In the comparation with other sites (Fig. 3), we observed that there are significant differences in the distribution of individuals with all assemblages according to the χ^2^ test, although the lowest χ^2^ values were found for the kill-butchery (Clary Ranch (χ^2^ = 14.618, *p* = 0.0234), Scottsbluff (χ^2^ = 15.43, *p* = 0.0171)), natural trap (Hawken (χ^2^ = 15.465, *p* = 0.0305), Kiputz IX (χ^2^ = 16.467, *p* = 0.0115)) and arroyo jump sites (Rourke (χ^2^ = 15.251, *p* = 0.0329)). In contrast, the largest differences are found at the Cordero Mine site (χ^2^ = 49.333, *p* < 0.000001), a processing area. These results are in agreement with the similarity analysis that was performed (Fig. 3B).

The estimated body mass of adult specimens (see Table 1) reflects that the mean value for the TD10.2-BB population was 410.72 kg (sd = 135.97), with specimens having a maximum value of 1028 and a minimum of 74 kg. It should be noted that only 37 out of 908 (< 5%) estimates are above 700 kg, most of these in estimates from the length of the M1. Considering the proportion of individuals in each age interval, we estimated the mean weight of individuals in the population (PBM) to be 270.97 kg (Table 2).

**Table 2:** Estimations of average body mass (kg) of the population from biomass values for each age interval, obtained from body mass and the relative proportion of individuals in each age interval (*P*_*i*_).

Age intervals (years)	Body mass (kg)	*P*_*i*_	Biomass per age class (kg)
0 (0–2)	135.49	0.40	54.65
1 (2–4)	316.26	0.28	87.70
2 (4–6)	393.08	0.13	49.55
3 (6–8)	407.87	0.10	41.13
4 (8–10)	410.27	0.05	20.69
5 (10–12)	410.65	0.02	6.90
6 (12–14)	410.71	0.02	6.90
7 (14–16)	410.72	0.01	3.45
8 (16–18)	410.72	0.00	0.00
9 (18–20)	410.72	0.00	0.00
			Mean = 270.97

## Discussion

The initial hypothesis of this study was whether the human groups exploiting the bison at the TD10.2-BB level of Gran Dolina did so in a sustainable manner. To this end, we set out to analyze whether the pattern of death of these bison could have been recurrent and allowed the bison to be exploited in the long term or, on the contrary, whether it could have led to the collapse of this population. Studies of small-scale societies that have communal hunting among their subsistence repertoire indicate that these events are sporadic, usually concentrated in one part of the year (seasonal), when resources are concentrated, more productive or more easily exploitable^2,6^. No group subsists solely based on daily communal hunting, this being a complement to other foraging strategies. Our results indicate that bison are recorded in all juvenile and adult age intervals except interval 5 (10–12 years) (Fig. 1). However, in the senescent phase there is no representation in the last two intervals (8 and 9, from 16 to 20 years), as the maximum longevity reached by the individuals in the sample analyzed is 16 years. The lack of representation in some age intervals does not seem to be due to preservation problems, as the sample from this population has a very high degree of preservation when comparing teeth from both hemimandibles (50 vs. 50, 100%). The mortality profile we have reconstructed conforms to catastrophic or life-structured mortality patterns, both by the ternary diagram analysis of Stiner^13^ (L-shaped) (Fig. 2A) and that of Discamps and Costamagno^14^ (JPO) (Fig. 2B). Regarding the ecological characteristics of this bison population, our results suggest that the population would be growing, since the net growth rate (*R*_*o*_) is greater than 1 (1.228) (Table 1), increasing the population by almost 10% annually. Thus, this mortality pattern would not lead to population collapse. Other ecological parameters of these bison are that they had an average fecundity rate of 0.58 and that class 5, the last class of the adult phase (10–12), had the highest survival rate. This mortality pattern and ecological characteristics of the TD10.2 bison population show no significant differences with the mortality profiles of reference populations proposed by Frison and Reher^15^ for a normal population and with those obtained by the Weibull model for stable and stationary populations (see^16,17^) (Fig. 1). The results of this analysis reinforce the view that these bison could have been exploited in an ecologically sustainable manner by humans at TD10.2-BB paleoecosystem. In addition, the χ^2^ comparison with taphocoenosis of sites with known origin shows that the bison from TD10.2-BB are closer to kill-butchery sites (Fig. 3), which is consistent with the proposal defended for this assemblage in previous research^9^. In summary, our results suggest that the humans occupying the Gran Dolina TD10.2-BB hunted bison in a sustainable manner, considering that communal hunting occurred seasonally, once or twice on a recurrent basis, and that the mortality pattern is consistent with the use of the cave as a kill-butchery site.

In order to gain a deeper understanding of the biological characteristics of the bison population from Gran Dolina level TD10.2-BB and the use that humans made of them, we have estimated the average body mass of the adult individuals of this group, obtaining a value of 411 kg. This average body mass is lower than that estimated for the large bovid (700 kg) from the Galería site^18^, in the Sierra de Atapuerca, with a dating and location close to the Gran Dolina level TD10.2, situated less than 100 m away. However, the estimated body mass is practically the same as that of the bison from the lower part of the Gran Dolina site (TD6 and TD8 units) (410 kg)^18^. This smaller size has been used to distinguish it from the bison of the more recent level TD10.1^19^. When considering sub-adult individuals, we obtain a population average body mass of 271 kg (see Table 2 and^12,20^), which shows the relevance of these individuals in the population structure.

From this 271 kg average for everyone in the population, we can infer how humans exploited it, calculating how much meat and kilocalories could be yielded from their bodies. It is estimated that between 50^21^ and 60%^22^ of the total mass of a bison can be extracted for consumption. Thus, from the average body mass of a TD10.2-BB individual of 271 kg, an amount of meat between 135 and 163 kg could be obtained. Today, one kg of American bison (*Bison bison*) meat yields 1220 kcal (74% water, 23.3% protein, 2.43% fat and 0.27% other nutrients)^23,24^. Assuming a similar composition between American and TD10.2 bison, humans could obtain from 135 and 163 kg between 165,294 and 198,353 kcal, respectively.

In addition to the biomass and energy extractable from bison, we can deepen the relationship between humans and bison by knowing how much meat the human groups of TD10.2-BB demanded. Humans from Gran Dolina level TD10.2-BB are considered to be pre-Neanderthal like those from the Sima de los Huesos, also from the Sierra de Atapuerca^25^. The daily energy demand of Neanderthals is the subject of hot scientific debate (e.g.,^26,27^). Venner^28^ proposed in his study that the energy demand of an adult Neanderthal was approximately 3450 kcal/day, a value obtained by refining previously used approaches that proposed a range of between 2870 and 6754 kcal/day^26,27^. This estimate is above the range shown by some current hunter-gatherer populations^29^, but close to those of Siberian Yakuts with moderate physical activity (≈ 3200 kcal/day)^28^. In many modern hunter-gatherer populations, consumption of animal resources is found to account for 30–60% of daily intake^29^. However, according to a dental wear analysis, El Zaatari et al.^30^ suggest that Neanderthal populations had different meat consumption according to the type of environment, covering more than 85% of their diets with meat in open environments, and between 64 and 54% in closed environments. In addition, they observed that the Neanderthal population of Monsempron 3 had a micro-wear similar to that of Khoe-San hunter-gatherers, whose diets contain between 60 and 80% plant matter, i.e., only between 20 and 40% meat in their diet (e.g.,^31^).

Assuming an equivalent expenditure between pre-Neanderthals and Neanderthals, an adult individual could demand 2,933 kcal of meat daily if the percentage of meat in the diet was 85%. Based on the energy intake values for TD10.2-BB bison, one bison (between 165,294 and 198,353 kcal, see above) could provide the daily meat requirement for 56–68 adult humans. In scenarios with lower meat demand (30 and 60%), the meat requirements of between 80 and 192 adult individuals could be covered. In modern hunter-gatherer populations, the optimal group size is approximately 30 individuals^32^. In the case of humans in the Sierra de Atapuerca sequence, Rodríguez-Gómez et al.^33^ obtained sustainable densities close to 0.2 ind/km^2^, so that a group of 30 individuals had to have a home range of 150 km^2^, which is in the second quartile of area groups used by recent hunter-gatherer populations (see^32^). Considering a group size of 30 adults with high meat demand (85%), a single bison could feed them for two days, and more than 6 days for a group with low meat demand (30%). We know that at level TD10.2-BB there are at least 60 bison that died mainly at two times of the year, between (i) late spring and early summer, and (ii) early autumn^9^. This number of bison could provide the meat needed by a human group of 30 individuals for a year if the consumption of this resource in the diet was approximately 26–32%. These values are considering all individuals of the human group as adults, considering that the demand of sub-adult individuals would be lower, the percentages of meat in the diet could be higher.

If the hunting events of this bison population were mainly concentrated at two times of the year, the amount of meat available to them could exceed the demands of the human groups. However, we must consider that the two events that have been recorded at minimum may not have occurred consecutively in the same year. It should be noted that humans have very acidic stomach pH values (1.5 on average), which overlap with those of obligate and facultative scavengers (1.3 ± 0.08 and 1.8 ± 0.27, respectively), and is lower than that of generalist carnivores (2.2 ± 0.44), omnivores (2.9 ± 0.33) and specialist carnivores (3.6 ± 0.51) (data from^34^). This pH value in humans makes it possible to cope with parasites and to be able to consume meat days after the animal is dead^34,35^. Based on ethnohistoric sources, other researchers indicate that the ancestral human microbiota was probably complex enough to allow eating meat, fish and fat in an advanced state of decomposition without problems, and that far from being rejected, putrid meat and fat were preferred by many small-scale societies historically^36,37^. In fact, Speth^36^ and Speth and Morin^37^ propose that "eating putrid meat and fat, early hominins could have acquired many of the benefits of cooking [pre-digestion], but at much lower cost, and quite likely long before they gained control of fire"^37^. However, we are limited in the amount of protein we can consume, having to be below 35% of calories consumed, due to the negative and potentially lethal consequences (rabbit starvation) of such high protein intakes^6,38^. These can only be sustained with high percentages of fat (65–75%) as occurs in populations such as Inuit, northern Athabaskans, Siberian hunter-gatherers, and other populations in Nordic environments^38,39^. If the protein metabolism of TD10.2 humans was like ours and they were limited in their protein intake, fat utilization could be essential. Indeed, taphonomic analysis of the bison bone assemblage from TD10.2-BB revealed intensive utilization of organs and fatty tissues, including the tongue, marrow, and intraskeletal fat. Additionally, evidence of deliberate fractures was observed in elements such as rib angles, proximal, and medial phalanges^9^ indicating an extreme exploitation of fat, always present in a lower proportion than in lean meat. This pattern is widespread across numerous Paleolithic sites^40,41^, indicating a common practice. Thus, for a daily energy demand of 3450 kcal, they would need between 2243 (65%) and 2588 (75%) of fat to consume large amounts of protein. Based on the composition of bison meat, a bison with 50% of its weight used (i.e., 136 kg of meat) would provide approximately 30,000 kcal of fat, which would be required by 13–15 people. Although these amounts of fat could be supplemented by fat-rich tissues such as bone marrow or brain, which could supply more than 3000 kcal^42,43^, if these populations were metabolically limited in their protein consumption, likely, they did not fully utilize bison carcasses, leaving an appreciable amount of meat resources, as Speth^6^ proposed for Paleoindian bison-hunting populations on the North American Great Plains. However, it must be also considered that the resources that could be found in the Sierra de Atapuerca during the Middle Pleistocene were abundant and diverse, being able to obtain proteins and fats from various animal and plant source^9,19,33,44,45^, which would reduce the problem of protein metabolism and allow a complete utilization of bison carcasses.

A key question arising from our results is whether the observed sustainability was intentional and therefore conscious, falling within what we would call "anthropic decisions," or whether it resulted from natural ecosystem dynamics. Today, there are management and conservation plans exist for large mammal populations that sometimes targeting specific population groups based on sex, age, or size^46^. The detection of such biases in the fossil record would make it possible to defend the intentionality of human behavior in the past. In the case of the TD10.2 bison exploitation, there are important empirical limitations in determining whether human populations consciously developed sustainable strategies or whether they were simply the result of natural dynamics. On the one hand, based on our results, we cannot support a preference for a specific age group and sex, as occurs in current population interventions^46^, because we did not observe a specific bias, but rather that humans hunted with the same preference across age classes in the mortality profile. On the other hand, regarding body mass, as mentioned above, we observed differences between the estimates for adult bison from TD10.2-BB (410.72 kg) and those from Galería (700 kg)^18^, which could lead us to consider a possible bias towards small-sized individuals, although it has been proposed that they are different species^19^. It should be considered that current European and American bison populations represent groups of more than 20 individuals in open ecosystems, with groups reaching 100 individuals in winter, and with different compositions: herds of males (less than 10 individuals), mixed herds (19–480 individuals), and herds of females with calves (10–63 individuals)^47–49^. Mass hunting involved the selection of groups or herds and based on the characteristics of TD10.2-BB in terms of mortality profile and individual size, we can assume that humans selected herds of females with calves. Thus, although we see no bias towards a particular age class, humans selected herds with a particular composition that would allow for population sustainability. Could we distinguish whether the selection of certain herds was a consciously evolved behavior to maintain bison populations, or whether it could be the result of natural dynamics? This is very complicated with the information we have. From an ecological perspective, there are mechanisms at the ecosystem and population levels that allow achieving stable dynamics. Behavioral ecology suggests that nonhuman predators evolve behaviors that inherently limit overexploitation (e.g.,^50^). These behaviors, including territoriality, selective hunting, and energetically efficient hunting methods, have been observed in large carnivores such as wolves and lions, where sustainable prey management naturally occurs, and in the case of humans, cultural constraints can be added to ecological constraints^51^. Archaeological evidence has shown that Neanderthal and pre-Neanderthal groups had a deep knowledge of the topography of the regions they inhabited and the behavior of animals, which may have allowed them to plan hunting strategies and territorial movements^52^. However, this does not necessarily mean that their behavior was consciously oriented towards ecological sustainability, especially concerning the population dynamics of their prey. In fact, overhunting of ungulates may have occurred, at least locally, during the Middle Paleolithic^53^. This is consistent with what we know about contemporary small-scale foraging societies, which can deplete resources locally while maintaining an overall balance with their environment^54,55^. It has been proposed that contemporary hunter-gatherer groups achieve sustainability with the environment as a secondary effect rather than as an intentional strategy, using the term epiphenomenal conservation^56^. Epiphenomenal conservation postulates that ecosystem sustainability or conservation is not the result of deliberate effort, but rather the inability of a population to overexploit resources or destabilize the environment in the long term^56,57^. If stable bison use at TD10.2-BB was simply the result of epiphenomenal conservation, it could have been driven by factors such as low population density, limited technological capabilities, or lower resource demand. However, we do not have sufficient information to defend the persistence of these factors for the humans who exploited bison at TD10.2-BB.

We know that human consumption of bison occurred in the Sierra de Atapuerca from the Early Pleistocene to the end of the Middle Pleistocene^9,19,58,59^. Different bison species (*Bison schoetensacki* and *Bison* sp.) are identified at the TD10 level of the Gran Dolina site^19^, and a relatively continuous sequence of human occupations is observed throughout its sequence, with technology evolving from the Acheulean (TD10.4 and TD10.3) to technologies indicative of early Mode 3 (TD10.2 and TD10.1)^60–63^. From the analysis of tooth eruption and wear patterns, Rodríguez-Hidalgo et al.^45^ argued that humans killed bison from level TD10.2-BB in two different seasonal windows (early spring and autumn). In a recent study of the archaeo-stratigraphy of level TD10.2, Arteaga-Brieba et al.^62^ have confirmed at least two hunting events in level TD10.2-BB, as well as different anthropic impacts both above (Upper TD10.2) and below (TD10.2.2), in which the dominance of bison is also evident, although not communal hunting. There is an apparent cultural continuity throughout the TD10.2 level, as the three archaeological sublevels (TD10.2.2, TD10.2BB, and TD10.2 Superior) present lithic technology with homogeneous characteristics^62^. This could support a continuous relationship through time between humans and bison, but higher resolution would be required. We believe that sustainable use of the TD10.2BB bison would not be a deliberate behavior but would result from a continuous relationship between bison and humans that have co-evolved and achieved a stable relationship between their populations. A record of continuous human activity over time in level TD10.2 of the same human groups could support our proposal. However, although bison consumption in the Sierra de Atapuerca and in level TD10 seems to be relatively continuous over time, the evidence obtained so far does not allow us to validate coevolution between bison and human populations. We hope that future analyses of the level will allow us to move in this direction.

## Conclusions

Our study suggests that human groups practicing communal hunting at the Gran Dolina level TD10.2-BB exploited bison sustainably. The mortality pattern of the bison population indicates growth rather than collapse, and its ecological characteristics are consistent with those of stable populations. The Gran Dolina TD10.2-BB bison population exhibited a mortality pattern consistent with sustainable exploitation, with high survival to adulthood and no significant deviation from reference mortality profiles. The average body mass of adult bison individuals allowed estimation of meat and kilocalorie yields, indicating substantial potential energy extraction from bison carcasses. Based on estimates of Neanderthal energy requirements and diet composition, bison meat could have met the daily meat requirements of a significant number of individuals in pre-Neanderthal human groups at TD10.2. Given the limitations on protein intake and the need for fat consumption for energy balance, it is proposed that bison carcasses may not have been fully utilized, leaving important meat resources available, especially if supplemented with fat-rich tissues. The availability of abundant and diverse resources in the Sierra de Atapuerca during the Middle Pleistocene probably reduced metabolic constraints on protein consumption, allowing the optimization of bison carcasses along with other animal and plant sources. The timing of hunting events, potentially concentrated in late spring and early summer as well as early fall, could have provided a surplus of meat beyond human demands, which could indicate separate hunting events throughout the year, groups much larger than those theoretically proposed for hunter-gatherer societies of the Middle Pleistocene or even a high waste of lean meat in each hunting event. Overall, the study suggests that human exploitation of bison at the Gran Dolina TD10.2-BB level was sustainable, supported by a combination of ecological, nutritional, and behavioral factors.

## Materials and methods

The Gran Dolina site, located in the Sierra de Atapuerca in northern Spain, is a karst cave that contains over 20 m of sedimentary infill. This sediment is divided into 11 lithostratigraphic units, labeled TD and numbered from the bottom to the top (from TD1 to TD11)^64^. The human occupation of the cave dates from 0.9 million to 0.2 million years ago and features rich deposits of lithic artifacts, anthropized fauna, and hominin remains^19,60^. Among the Middle Pleistocene deposits, the TD10 lithostratigraphic unit, which is about 3 m thick, is notable for its variety of anthropic occupations. Studies focusing on archaeostratigraphic, spatial analysis, and lithic refitting have revealed various human activities, ranging from isolated instances of tool-making or animal carcass processing to extensive bone beds. These bone beds cover the entire excavation surface of approximately 80 m^2^ and can be several centimeters thick, containing tens of thousands of archaeological remains^9,60,62,63,65,66^.

Gran Dolina acted as a base camp for Middle Pleistocene hominins, especially in the TD10.1 bone bed layer^61,65^, with a series of lower-intensity cumulative events observed in Upper TD10.1, TD10.3, and TD10.4^60,63,66^.

In the lithostratigraphic subunit TD10.2, a bone bed approximately 20 cm thick was identified both in the field and later archaeostratigraphically, containing over 40,000 faunal remains and 9000 lithic artifacts, referred to as the TD10.2 bison bone bed (TD10.2-BB)^60,62,66^. Analysis of a sample of 24,216 faunal remains indicates a moderate to high variety of taxa (n = 18), but bison remains represent 98.4% of the Number of Identified Specimens (NISP = 22,889) and the 99% of the ungulates NISP^9^. Thus, the TD10.2 bison bone bed can be considered monospecific (Shannon Evenness Index = 0.02; Simpson’s Index (D) = 0.009). The taxa related to ungulates other than *Bison* sp. in the assemblage total 107 remains, which is 0.4% of the NISP (exclusively equids and cervids). Furthermore, no anthropogenic marks were found on the remains of these animals, while there are more than a thousand bison remains with anthropogenic marks, which has been interpreted to indicate a non-anthropogenic origin for ungulates other than bison. The remaining animals are large and small carnivores, birds, rodents, and lagomorphs, also unrelated to the anthropogenic component. The detailed and extensive zooarchaeology and taphonomic studies published by Rodriguez-Hidalgo et al.^9,45,62^ indicate short but highly intensive, overlapping occupations (at least two), suggesting that hominin groups who hunted and processed at least 60 (see^45^) bison carcasses used the cave as a kill-butchering site. Analysis of bison mortality profiles, tooth eruption and wear, seasonality inferred from dental microwear, and evidence of intensive human processing, along with selective transport and abandonment of carcass parts, indicate that bison were mass hunted and exploited during at least two communal drives. Processing was intensive, with butchering marks indicating extensive exploitation of meat, internal organs, tongue, and especially marrow, including from low-yield elements like mandibles, phalanges, and ribs^9,65^. The intense human activity recorded, followed by intense carnivore ravaging, makes the assemblage highly fragmentary, with few complete bones, and complete epiphyses. These taphonomic characteristics, combined with the scarcity of appendicular bones in the assemblage, attributed to human transport, have resulted in an assemblage in which it is difficult to estimate the sex or size of bison exclusively from postcranial elements. However, dental remains are abundant and very well preserved, and no sexual dimorphism is evident.

The lithic industry, classified as early Mode 3, is characterized by a significant amount of in situ knapping primarily aimed at flake production^60,62^. The TD10.2 lithostratigraphic subunit, which is approximately 50 cm thick, has been dated to 378 ± 10 ka and 375 ± 37 ka^67^, 418 ± 63 ka and 337 ± 51 ka^68^, as well as 244 ± 26 ka^69^. The archaeological level TD10.2-BB falls within this range of geochronological uncertainty.

In this study we use fossil bison materials from TD10.2-BB in order to reconstruct the mortality profile and life table of this population, on the one hand, and to estimate their body mass, on the other hand. In the reconstruction of the mortality profile and life table of the TD10.2 bison population, we used dental remains to estimate the ages of individuals. For this, we used the approach of Klein and Cruz-Uribe^70^ in which crown heights and tooth eruption ages are used. We selected lower D_4_ and P_4_ (see Table SI1) as the most frequent remains, to reduce the chances of considering the same individual several times, having a total of 50 specimens, 21 D_4_ (10 right and 11 left) and 29 P_4_ (15 right and 14 left) (25 right specimens *versus* 25 left). According to these proportions, conservation is very high. When we obtained the same values for specimens from different sides of the hemimandible, we considered them to be the same individual to avoid multiple counting. Thus, the total number of individuals available to reconstruct the mortality profile and life table of the TD10.2 bison was 48 individuals, i.e., we did not include two specimens. Age estimation according to the method of Klein and Cruz-Uribe^70^ requires several inputs of information that are in part specific to the fossil population, such as the height of the crown of the deciduous and permanent tooth without wear, and others that must be inferred, such as the age of eruption of the permanent tooth or the maximum longevity. For a deciduous tooth:

$${\text{AGE}} = {\text{AGE}}_{{\text{S}}} - {\text{2AGE}}_{{\text{S}}} \left( {{\text{CH}}/{\text{CH}}_{0} } \right) + {\text{AGE}}_{{\text{S}}} \left( {{\text{CH}}^{{2}} /{\text{CH}}_{0}^{{2}} } \right)$$

For a permanent tooth:

$${\text{AGE}} = {\text{AGE}}_{{{\text{pel}}}} - {2}\left( {{\text{AGE}}_{{{\text{pel}}}} - {\text{AGE}}_{{\text{e}}} } \right)\left( {{\text{CH}}/{\text{CH}}_{0} } \right) + \left( {{\text{AGE}}_{{{\text{pel}}}} - {\text{ AGE}}_{{\text{e}}} } \right)\left( {{\text{CH}}^{{2}} /{\text{CH}}_{0}^{{2}} } \right),$$

where CH is the crown height, CH_0_ is the initial crown height, i.e., unworn, AGE_S_ is the age at which a deciduous tooth is lost, AGE_e_ is the age at which a permanent tooth erupts, and AGE_pel_ is the maximum possible age of an individual, i.e., the age of potential ecological longevity. Crown height (CH) was measured as the minimum distance between the occlusal surface and the line separating the crown enamel from the root dentin (crown-root junction). As these were mandibular teeth, measurements were made on the buccal surface of the anterior lobe according to Klein and Cruz-Uribe^70^. According to Wegrzyn and Serwatka^71^, the age at which the lower P_4_ replaces the D_4_ (AGE_e_ and AGE_S_, respectively) in European bison (*Bison bonasus*) is approximately 3 years of age, we use this value to fix this replacement in the bison of TD10.2 and take the D_4_ with the highest wear as the time of tooth replacement. Regarding maximum ecological longevity, we use as a reference the work of Discamps and Costamagno^14^, in which they use a value of 20 years for bison. This longevity value is close to that observed for European bison and similar to that of other bovids, as can be observed in Table 3. As part of the reconstruction of the population profile, we defined the age classes up to maximum longevity in two-year intervals, reducing the chances of erroneously assigning individuals to an age class, and distributed the 48 individuals according to the calculated age. This distribution is the basis for inferring aspects about the ecology of the TD10.2 bison population that we will obtain from the life table.

**Table 3:** The life-history traits of living bovid species used to estimate those of the TD10.2-BB bison from three database: PanTHERIA^78^(^a^), AnAge^79^(^b^) and Animal Diversity Web^80^(^c^).

Species	ABM	NBM	AFB	LS	LY	L
*Bison bison*	625^a^	20.00^a^	3.00^a^	0.98^a^	0.90^a^	18^c^
*Bison bonasus*	676^a^	23.36^a^	2.93^a^	1.00^a^	0.57^a^	19^c^
*Bos frontalis*	800^a^	22.98^a^	2.42^a^	1.22^a^	1.10^a^	26^c^
*Bos grunniens*	667^b^	18.00^b^	2.82^a^	1.00^a^	0.80^b^	25^c^
*Bos javanicus*	700^b^	–	2.50^a^	1.00^b^	1.00^a^	18^c^
*Bos sauveli*	796^c^	–	–	1.00^b^	1.00^c^	20^c^
*Bos taurus*	750^b^	–	–	1.00^b^	1.00^a^	20^c^
*Boselaphus tragocamelus*	182^a^	5.88^a^	2.50^a^	1.39^a^	1.05^a^	21^c^
*Bubalus bubalis*	1000^c^	37.50^a^	1.50^c^	1.00^c^	0.50^c^	25^c^
*Bubalus depressicornis*	155^b^	-	2.00^c^	1.00^a^	1.00^a^	20^c^
*Bubalus mindorensis*	240^c^	-	2.00^c^	1.00^a^	0.50^c^	20^c^
*Bubalus quarlesi*	182^a^	–	2.00^c^	1.00^a^	1.00^a^	29^c^
*Pseudoryx nghetinhensis*	90^c^	–	–	1.00^c^	1.00^c^	18^c^
*Syncerus caffer*	593^a^	40^a^	4.00^a^	1.08^a^	0.40^b^	22^c^
*Taurotragus derbianus*	680^b^	–	3.03^b^	1.00^b^	–	20^c^
*Taurotragus oryx*	563^a^	30.89^a^	1.61^b^	1.02^c^	1.10^b^	24^c^
*Tragelaphus scriptus*	43^a^	3.03^a^	1.36^c^	1.00^a^	1.55^b^	15^c^
*Tragelaphus spekii*	88^c^	4.00^a^	1.10^b^	1.00^a^	0.97^c^	23^c^
*Tragelaphus strepsiceros*	218^b^	15.37^a^	1.42^b^	1.00^a^	1.00^b^	23^c^
*Tragelaphus angasii*	120^b^	5.54^a^	1.24^b^	1.00^a^	1.24^b^	19^c^
*Tragelaphus buxtoni*	215^a^	–	1.75^c^	1.00^a^	–	18^c^
*Tragelaphus eurycerus*	308^b^	19.94^a^	2.21^b^	1.00^a^	0.70^b^	22^c^
*Tragelaphus imberbis*	83^b^	5.89^a^	1.38^b^	1.00^a^	1.41^b^	19^a^
TD10.2-BB bison	411	18.48	2.21	1.00	0.89	20

*ABM* adult body mass (in kg), *NBM* neonate body mass (in kg), *AFB* age at first birth (in years), *LS* litter size, *LY* litters per year, *L* longevity (in years). The cells show a dash when there are no values for some variables.

In life table analyses, only female demographic parameters are considered when applying this tool to mammal populations^72^ as it is assumed that the survival rate of males does not affect the growth rate of most populations^73^. Thus, the reproductive rate per age interval is divided by 2 to consider only female individuals as we assume an equilibrium sex ratio at birth, i.e., 50% for males and females (1:1)^74,75^. Furthermore, we also assume that the age structure is stable and stationary over time, with constant survival and mortality rates for different cohorts, and that the age structure recorded at the site corresponds to a local population in which migratory flows did not modify the assemblage^76^. It is worth noting that the assumptions to be considered in this paper are not overcome in work on current large mammal populations because of the difficulty of monitoring entire cohorts over long periods of time^74,77^.

Life tables are composed of different demographic parameters (see^75^), where: "*x*" is the age interval, which in the present work each one spans two years, starting at interval 0 and ending at interval 9. For instance, any individual with an age value between 0 and 24 months is integrated in the age interval 0 (*x*_*0*_). The second age interval (*x*_*1*_) corresponds to individuals with an estimated age between 24 and 48 months (2 and 4 years), and so on. "*d*_*x*_" is the proportion of individuals who die between age x and x + 1: *d*_*x*_ = *l*_*x−* _−* l*_*x*+*1*_; "* l*_*x*_ " is the proportion of individuals in the cohort who survive in an age class: *l*_*x*+1_ = (*l*_*x*_*—d*_*x*_), generally the initial value can be 1 (as in this study) (Table 1), 100 or 1000 in the first age interval; "*q*_*x*_" is the interval-to-interval mortality rate: *q*_*x*_ = *d*_*x*_/*l*_*x*_; " *s*_*x*_ " is the survival rate of an age class to the following: *s*_*x*_ = *l*_*x*+*1*_/*l*_*x*_; "*m*_*x*_" is the fecundity rate, number of offspring per female in each interval. For the bison of TD10.2 we do not know their fertility values for each age class (*m*_*x*_), for this reason, we used data provided by different databases for current populations of species of the subfamily Bovinae^78–80^ (Table 3). From the values of litter size (LS), number of litters per year (LY), inter-litter interval, sexual maturity of females, and the fertility period of the species of the subfamily (12 years)^80^ we obtain regressions that relate these different variables to body mass and from which we can infer the values of the TD10.2 bison population at Gran Dolina (see Tables 3 and 4). In the case of litter size, we take the median value, which is 1 calf per litter. "*R*_*0*_" is the net reproduction rate of the cohort and corresponds to the average reproductive success of the population, *R*_*0*_ = *Σ l*_x_*m_*x*_. When *R*_*0*_ is less than 1 the population decreases while when *R*_*0*_ is greater than 1 it grows, being stable when it is equal to 1. We also use the mean generation time "*T*" which gives the average interval between the birth of an individual and the birth of its offspring. The age of the individuals (*x*) is multiplied by the proportion of individuals surviving to this age (*l*_*x*_) and by the average number of offspring at this age (*m*_*x*_). This index is computed for each age interval, all values are summed and divided by (*R*_*0*_): *T* = ∑ x*l_x_*m_*x*_/*R*_*0*_. From the net reproductive rate (*R*_*0*_) and the mean generation time (*T*) we obtain lambda or the asymptotic growth rate (*λ*) (*λ* = *anti-ln (ln(R*_*0*_*)/T)*) because not all females reach maximum longevity.

**Table 4:** Allometric equations obtained for the different variables from regressions of the values in Table 3.

	Equations	R^2^
Neonate body mass (NBM)	NBM = 0.1501*ABM^0.7998^	0.87
Age at first birth (AFB)	AFB = 0.4559*ABM^0.2615^	0.45
Litters per year (LY)	LY = − 0.174*ln (ABM) + 1.9349	0.33

*ABM* adult body mass.

The mortality profile of the TD10.2-BB bison obtained from the age estimates of their remains allows us to estimate the mortality pattern using the ternary diagrams defined by Greenfield^81^, and further modified by Stiner^13^ and by Discamps and Costamagno^14^. The two-year age intervals that were used to classify individuals according to age estimates were used for grouping into life-cycle stages. In this paper we use the same values as Discamps and Costamagno^14^ for bison (juveniles: 0–3 years [0–36] months; prime adults: 3–12 years [36–144] months; old adult: 12–20 years [144–240] months. In the case of the juvenile phase, the age interval does not coincide with the limits of the age intervals, but we selected those individuals with age estimates below 3 years, which are those with D_4_ and which had not yet been replaced by P_4_ (see^71^).

In order to analyze the population dynamics of the TD10.2-BB bison, we compared the mortality profile obtained in our study with reference bison mortality profiles, such as that of Frison and Reher^15^. In the work of Frison and Reher^15^, values are given for the frequency of individuals by age interval, giving a value for half of each year. We grouped these values in two-year intervals to make them comparable with those obtained in the present study. On the other hand, we compare TD10.2 values with stable and stationary structures obtained from fertility parameters and the application of the Weibull model, according to the approximation of Martín-González et al.^16,17^. Martín-González et al.^16,17^ studied several models to reconstruct age structures of fossil populations, and the best fitting model was the Weibull model, combined with population dynamics based on Leslie matrices. Martín-González et al.^16^ established stability and sustainability conditions in the Weibull model to obtain age structures for large mammal species that were sustainable in the long term, on the understanding that they represent the average of population fluctuations (see^82^). To apply this approximation, we used the inferred fertility values for bison from TD10.2 (see Table 3). The Weibull model provides many results for both survival and mortality profiles. In this paper, we selected the extreme structures of survival (minimum (_m_S) and maximum (_M_S), respectively) and mortality (maximum (_M_M) and minimum (_m_M), respectively) profiles. Finally, we compare the TD10.2 mortality profile with values from sites where bison accumulation of natural and anthropogenic origin is proposed (see Table SI2). In order to compare the different profiles with the one obtained for the TD10.2 bison, we grouped the individuals by comparable age intervals, reconstructed the profiles with the same number of individuals as in TD10.2 (48 individuals), and performed a χ^2^ test, a dendrograma, and a Bray–Curtis similarity analysis.

In addition to reconstructing the ecological characteristics of the bison population using life tables, we estimated the body mass of this population to understand its physical characteristics. In this work, we use both cranial and postcranial remains in order to have as many estimates as possible, although we are aware that cranial remains provide less precise values (e.g.,^83^). The postcranial remains used were: humeri (1 specimen), radii (3), tibiae (3), metacarpus (8), metatarsus (4), and astragalus (2); and as cranial remains different dental measurements from P_2_ (82), P_3_ (82), P_4_ (45), M_1_ (22), M_2_ (144), and M_3_ (61) (see Table 5 and Table SI3). The estimated body masses were found using different allometric equations. For limb bones, we used the Scott^84^ equations for ruminants (in^83^ Table 16.7) (Table 5), for astragalus, those of the Family Bovidae of Rodríguez^18^ and for dental remains the equations of Damuth (in^83^ Table 16.9). We discard equations with percentage prediction error (%PE) values greater than 100%. When different estimates are obtained from the same specimen, we treat each of these estimates independently. We estimated the average weight of individuals in the population by weighting according to the %PE of the estimate, following to Filippini et al.^85^, so that the equations with the highest predictive ability would have the most weight. From this average value for adult individuals, we calculate the average body mass of the individuals in the population. Measurements were taken according to the Driesch^86^ model using a digital caliper to the tenth of a millimeter. The selected bones belonged to adult specimens (with fused epiphyses), as the equations are developed for them. For this reason, infant and juvenile specimens were discarded.

**Table 5:** Values of the equations used to calculate body mass of TD10.2-BB *Bison* sp. from Gran Dolina site.

Bones or teeth	Variables	Slope (b)	Intercept (c)	r^2^	%PE
Humerus^a^	Hm4	2.5499	0.4078	0.96	18
Radius^a^	Rd4	2.431	0.374	0.95	19
Tibia^a^	T5	2.972	0.6222	0.95	22
Metacarpus^a^	Mc2	2.6495	0.6016	0.95	20
Metacarpus^a^	Mc3	2.8291	1.0620	0.94	22
Metacarpus^a^	Mc4	2.3765	0.7443	0.92	25
Metatarsus^a^	Mt2	2.9220	0.6162	0.94	24
Metatarsus^a^	Mt3	3.0306	0.5755	0.93	26
Metatarsus^a^	Mt4	2.7421	0.5614	0.94	22
Metatarsus^a^	Mt5	2.9763	1.1416	0.92	28
Astragalus^b^	ASTDT	2.8760	0.9112	0.93	27
Astragalus^b^	ASTL	3.1192	− 0.1298	0.94	26
M_1_^c^	DVL	3.0400	1.8600	0.84	40
M_1_^c^	DMD	3.1300	1.2700	0.85	39
M_1_^c^	Area	1.5800	1.4800	0.87	35
M_2_^c^	DVL	2.8500	2.0000	0.80	43
M_2_^c^	DMD	3.1000	1.0500	0.85	39
M_2_^c^	Area	1.5300	1.4300	0.86	37
M_3_^c^	DVL	3.0400	1.8600	0.84	43
M_3_^c^	DMD	3.1500	0.5600	0.86	36
M_3_^c^	Area	1.5600	1.1600	0.87	35
P_4_^c^	DVL	2.5800	2.5600	0.82	44
P_4_^c^	DMD	3.1100	1.5000	0.80	47
P_4_^c^	Area	1.4600	1.9900	0.84	42
P_3_^c^	DVL	2.4700	2.8300	0.78	51
P_3_^c^	DMD	2.6400	2.1500	0.70	62
P_2_^c^	DVL	2.5700	3.0400	0.76	53

These equations were derived from single limb bones in bovids by Scott^84^ (^a^) and Rodríguez^18^ (^b^) and tooth specimens by Damuth and Macfadden^83^ (^c^) for selenodont non-browsers. Hm4: transversal diameter of distal articular surface in anterior view of the humerus; Rd4: maximum transversal diameter of proximal epiphysis of the radius; T5: anteroposterior diameter of distal epiphysis of the tibia; Mc2: transversal diameter of proximal articular surface of the metacarpus; Mt4: maximum transversal diameter of distal epiphysis of the metatarsus; ASTDT: maximum proximal transversal diameter of the astragalus; ASTL: astragalus length; DMD: mesio-distal diameter in tooth; DVL: vestibulo-lingual diameter in tooth. The equation has this form: log (*BM*) = b (log *X*) + c, where BM is the body mass (kg for Scott’s equations and g for Damuth’s and Rodríguez’s equations) and *X* is the measure of bones and tooth (cm for Scott’s equations and mm for Damuth’s and Rodríguez’s equations) in each case.

For the calculation of the average body mass of the population, we have followed the proposal of Rodríguez-Gómez. et al.^87^:

1 $$B = \sum {(P_{i} * \, M_{i} * \, D),}$$

where *B* is the biomass of the population, *P*_*i*_ is the relative proportion of individuals in the population in class *i*, and overall represents the population age structure, *M*_*i*_ is the average body mass of the individuals of class *i* and *D* is the density of the population, which in this analysis is equal to 1 since we want to know the average weight of the population. Therefore, the proportion of individuals (*P*_*i*_) and the average weight (*M*_*i*_) in each age class must be estimated. To calculate the relative proportion of each age class (*P*_*i*_), we used:

2 $$P_{i} = \frac{{l_{i} }}{{\sum {l_{i} } }},$$

where *l*_*i*_ is the proportion of individuals in the cohort who survive in class *i*. We obtained the *l*_*i*_ values for estimating *P*_*i*_ from the life table that we reconstructed for the TD10.2 bison from Gran Dolina (see Result section and Table 1).

To estimate body mass for all age intervals, from birth to adult size, we have used the approximation of Zullinger et al.^88^:

3 $$M \left(t\right)=ABM*{e}^{-{e}^{-K\left(t-I\right)}},$$

where *ABM* is the asymptotic mass (i.e., the adult body mass), *M (t)* is the mass (g) at age *t, K* is the growth rate constant (days^-1^), and *I* is the age at the inflection point (443.3 days). *K* is related to the mass of adults by the equation:

4 $${\text{log }}(K) = {-} \, 0.{9}0{1}{-}0.{3}0{2}*{\text{log }}(ABM)$$

The average body masses of the age intervals are calculated by taking the extreme values of each interval. In the first age interval, the average body mass between that of the new-born and that which is reached at two years of age is taken.

## Electronic supplementary material

Below is the link to the electronic supplementary material.

Supplementary Material 1

## Data Availability

The data sets generated and/or analyzed during the current study are available from the corresponding author upon reasonable request. Most of these data are included in the Supplementary Information.
